# Assessment of left ventricular preload by cardiac magnetic resonance imaging predicts exercise capacity in adult operated tetralogy of Fallot: a retrospective study

**DOI:** 10.1186/1471-2261-14-122

**Published:** 2014-09-23

**Authors:** Jonathan Yap, Ju Le Tan, Thu Thao Le, Fei Gao, Liang Zhong, Reginald Liew, Swee Yaw Tan, Ru San Tan

**Affiliations:** Department of Cardiology, National Heart Centre Singapore, 5 Hospital Drive, Singapore, 169609 Singapore; Duke-NUS Graduate Medical School, Singapore, 8 College Rd, Singapore, 169857 Singapore

**Keywords:** Exercise capacity, Cardiac magnetic resonance imaging, Tetralogy of Fallot

## Abstract

**Background:**

The optimal timing of pulmonary homograft valve replacement (PVR) is uncertain. Cardiopulmonary exercise testing (CPET) and cardiac magnetic resonance (CMR) are often used to guide the clinical decision for PVR in operated tetralogy of Fallot (TOF) patients with significant pulmonary regurgitation (PR). We aim to study the relationship between exercise capacity and CMR in these patients.

**Methods:**

The study is a single-centre retrospective analysis of 36 operated TOF patients [median 21.4 (interquartile range 16.4, 26.4) years post-repair; 30 NYHA I, 6 NYHA II; median age 25.2 (interquartile range 19.5-31.7) years, 29 males] with significant PR on CMR who underwent CPET within 15 [median 2.0 (interquartile range 0.8-7.2)] months from CMR. CPET parameters were compared with 30 age- and sex-matched healthy controls [median age 27.8 (interquartile range 21.0-32.8) years; 24 males].

**Results:**

Peak systolic blood pressure (177 versus 192 mmHg, p = 0.007), Mets (7.3 versus 9.9, p < 0.001), peak oxygen consumption (VO_2_max) (29.2 versus 34.5 ml/kg/min, p < 0.001) and peak oxygen pulse (11.0 versus 13.7 ml/beat, p = 0.003) were significantly lower in TOF group versus control. Univariate analyses showed negative correlation between PR fraction and anaerobic threshold. There was a positive correlation between indexed left (LV) and right (RV) ventricular end-diastolic volumes, as well as indexed LV and effective RV stroke volumes, on CMR and VO_2_max and Mets achieved on CPET. These remained significant after adjustment for age and sex.

**Conclusions:**

TOF subjects have near normal exercise capacity but significantly lower Mets, VO_2_max and peak oygen pulse achieved compared to controls. Increased PR fraction in TOF subjects was associated with lower anaerobic threshold. Higher indexed effective RV stroke volume, a measure of LV preload, was associated with higher VO_2_max and Mets achieved, and may potentially be used as a predictor of exercise capacity.

## Background

Long-term outcomes of patients with tetralogy of Fallot (TOF) have improved with advances in surgical techniques [1]. However, pulmonary regurgitation (PR) resulting from repair to the right ventricular outflow tract is a common consequence of the primary surgery. This is usually well tolerated initially, but leads to increased right ventricular (RV) dilatation, dysfunction and worsening exercise intolerance over time, and may result in arrhythmia and sudden cardiac death [2–4].

The optimal timing of pulmonary homograft valve replacement (PVR) in operated TOF patients with significant PR is unclear. The decision for PVR is usually a fine balance between avoiding permanent remodeling of the right ventricle [5, 6] and reducing the need for repeat procedures throughout one’s lifetime in these often young patients. Cardiopulmonary exercise testing (CPET) and cardiac magnetic resonance (CMR) are two important tools often used by clinicians to help guide this difficult management decision [5, 6].

Poor exercise capacity identifies patients at higher risk of hospitalization and mortality [7–9] and guidelines have listed decreasing exercise tolerance as a strong indication for PVR [10–12]. The American College of Cardiology/American Heart Association (ACC/AHA) 2008 guidelines recommend PVR as a Class I indication in operated TOF patients with severe PR and decreased exercise tolerance [12]; exercise testing is a useful adjunct. A Class IIa indication for PVR is in patients with severe PR and moderate to severe RV enlargement [12]. The exact definition of moderate to severe RV dysfunction/enlargement is not clear. The European Society of Cardiology (ESC) 2010 guidelines have similar recommendations but suggest using a RVEDVi cut-off of 160 ml/m2, beyond which normalization of RV becomes unlikely [10]. The Canadian Cardiovascular Society (CCS) 2009 guidelines recommend as a Class IIa indication that PVR should be considered in TOF patients with severe PR who have either a deterioriating exercise performance or moderate to severe RV enlargement (RVEDVi >170 ml/m^2^) [11]. A RVEDVi cut-off of 150 ml/m2 is sometimes used clinically [13]. A recent study found similar exercise capacity and stroke volumes in patients below and above the threshold, thus raising questions about the utility of RVEDVi as the main basis for deciding on timing for PVR [14].

The relationship between CMR parameters and actual exercise capacity remains unclear. Understanding the relationship between exercise capacity and CMR parameters is important and may better help rationalize the clinical decision for PVR. We aim to study the exercise capacity in TOF patients with significant PR late (>10 years) after repair and its relationship with CMR parameters.

## Methods

### Study design and patient population

In our institution, operated adult TOF patients with significant (at least moderate) PR on TTE will undergo CMR and CPET as part of their clinical management. The study is a retrospective review of all consecutive patients with surgically corrected TOF and known significant (moderate to severe) PR on TTE, who underwent CMR and CPET at our institution from Apr 2005 to Mar 2013. All patients were older than 15 years of age and had undergone corrective surgery for TOF more than 10 years previously [median 21.4 years (IQR 16.4, 26.4)]. Patients with residual pulmonary stenosis pressure gradient on Doppler echocardiography, associated atrioventricular septum defect, double outlet right ventricle of Fallot type, pulmonary atresia with ventricular septum defect, TOF with absent pulmonary valve, or patients who had undergone pulmonary valve replacement (PVR) were excluded. Clinical data were obtained from review of patients’ case-files and electronic medical records.

CMR and CPET data were recorded. CPET data were also obtained from age- and sex-matched healthy controls. The study was approved by the SingHealth Centralised Institutional Review Board (SingHealth, Singapore).

### Cardiac MRI

Study subjects underwent CMR on a 1.5 T Avanto scanner (Siemens). In each patient, fast imaging in steady-state precession (trueFISP) cine MR images were acquired in the left ventricular (LV) long axes (2-, 3- and 4-chamber views), RV outflow tract (RVOT) (oblique sagittal and coronal) planes as well as a parallel contiguous stack of 8 mm ventricular short-axis cine slices. Retrospective ECG-triggering was employed, achieving typical frame rates of about 25 per cardiac cycle. Throughplane flow measurements in planes across the aortic root and main pulmonary trunk were performed using velocity-encoded gradient-echo pulse sequences.

Calculation of left and right ventricular end-diastolic (EDV) and end-systolic (ESV) volumes, LV mass and ejection fractions (EF) was performed using CMRTools (London, UK). All volumes and LV mass measurements were then indexed to body surface area. The presence of RVOT aneurysm, defined as akinesia or dyskinesia (outward movement) during systole of part of the RVOT assessed from the basal ventricular short-axis cine images, was recorded. PR fraction (diastolic reverse flow expressed as a percentage of forward flow) was assessed from anterograde and retrograde flow in the main pulmonary artery (ARGUS software, Siemens, Erlangen). Effective right ventricular stroke volume (RVSV) was calculated as the difference between the total RV forward flow and the pulmonary regurgitant flow [15, 16]. Restrictive RV physiology was ascertained by presence of end-diastolic forward pulmonary flow, consistent with severely limited RV capacitance during right atrial contraction.

### Cardiopulmonary exercise testing

Cardiopulmonary exercise tests were performed on a Hewlett-Packard Cosmos treadmill machine. A maximal ramp protocol with fixed treadmill speed but constantly increasing incline gradient to provide an increasing workload was employed. Exercise speed was determined at the performing physician's discretion. Patients were encouraged to exercise until symptoms were intolerable, i.e. when Rating of Perceived Exertion reached 18/20. Heart rate was monitored continuously, and non-invasive blood pressure was taken every 2 minutes with both manual and automatic blood pressure. Oxygen saturation was monitored by a finger probe pulse oximeter. Oxygen uptake (VO_2_), minute ventilation (VE), and carbon dioxide output (VCO_2_) were measured breath by breath. The anaerobic threshold (AT) was chosen as the VO_2_ at which respiratory exchange ratio reached 1.0 and when there was a sudden and sustained precipitous rise in VCO_2_. Peak VO_2_ was defined as the value of averaged data during the final 15 seconds of exercise. The VE/VCO_2_ slope was determined as the linear regression slope of VE and VCO_2_.

### Statistical analysis

Data was analysed using STATA version 11.1 (College Station, Texas, USA). All values were presented as median and interquartile range for continuous data and frequency and percentage for categorical data. Comparisons between groups were made with Mann–Whitney U test, or the Fisher’s exact test as appropriate. The linear correlation, *r*, between exercise testing and CMR parameters were initially assessed using Pearson correlation coefficient with 95% confidence interval (CI). Significant relationships were subsequently assessed by multiple linear regression technique adjusting for age and gender. Scatter plots with linear fit were produced. For all analyses, a two-tailed *P* value of <0.05 was considered significant.

## Results

### Baseline clinical characteristics

Of the 119 TOF patients on clinical follow-up at our institution at the beginning of the study, 36 had significant PR on echocardiography. The latter underwent CPET and CMR testing and were included in this study. Thirty controls were also recruited. There was no significant difference in age, sex and indexed body mass between patients and controls. Six TOF patients had NYHA II functional status; the rest of the study TOF population were either asymptomatic or NYHA I (Table 1).

**Table 1:** **Demographics and CPET parameters of study population (n = 66)**

	TOF (n = 36)	Control (n = 30)	***P value***	Adjusted P value*
**Demographics**				
Age	25.2 (19.5, 31.7)	27.8 (21.0, 32.8)	0.20	
Male	29 (80.6)	24 (80.0)	1.00	
BMI (kg/m2)	22.8 (21.0, 26.3)	23.2 (19.6, 25.6)	0.39	
Asymptomatic/NYHA I	30 (83.3)	30 (100.0)	0.03	
**CPET parameters**				
Max HR (beats/min)	172.5 (159, 178)	176.5 (168, 181)	0.257	0.429
Heart rate reserve (beats/min)	26.5 (8, 39)	15.5 (7, 20)	0.117	0.183
Max SBP (mmHg)	177 (150, 184)	192 (168, 210)	**0.007**	**0.010**
Max DBP (mmHg)	86 (78, 93)	82 (76, 97)	0.575	0.964
Max breathing reserve (L/min)	38 (21, 72.1)	47.8 (34.5, 61.3)	0.540	0.602
Mets	8.3 (7.1, 9.4)	9.9 (8.6,11.7)	**<0.001**	**<0.001**
% predicted Mets (%)	85.0 (74.8, 93.9)	102.7 (91.1, 114.5)	**<0.001**	**<0.001**
Peak oxygen consumption (ml/kg/min)	29.2 (25.5, 33.0)	34.5 (30.0, 41.0)	**<0.001**	**<0.001**
% predicted peak oxygen consumption (%)	83.0 (72.0, 93.5)	102.7 (91.1, 114.8)	**<0.001**	**<0.001**
Anaerobic threshold (ml/min)	1395 (1170, 1574)	1501.5 (1255, 1700)	0.231	0.211
Oxygen pulse pressure (ml/beat)	11.0 (8.8, 13.2)	13.7 (11.9, 16.2)	**0.003**	**0.002**
VE/VCO_2_ slope	26.0 (23.6, 27.3)	25.6 (24.1, 27.2)	1.00	0.576
Arrhythmias**	7 (19.4)	4 (13.3)	0.742	0.492

Median and interquartile ranges are reported for continuous data and frequency and percentage for categorical data.

*Adjusted for age and gender.

**All were occasional ventricular or atrial ectopics.

### CPET parameters

Compared to controls, TOF patients achieved significantly lower maximum systolic blood pressure (177 versus 192 mmHg, p = 0.007), Mets (7.3 versus 9.9, p < 0.001), peak oxygen consumption (29.2 versus 34.5 ml/kg/min, p < 0.001) and peak oxygen pulse pressure (11.0 versus 13.7 ml/beat, p = 0.003). These differences were significant even after adjusting for age and sex. There were no significant difference in the rest of the CPET parameters (Table 1). 7 TOF patients experienced arrhythmia during CPET, all consisting of occasional isolated premature ventricular complexes. No malignant arryhtmia was noted.

### Medication, Surgical and CMR findings in TOF patients

The majority of the TOF patients were not on any cardiac medications: one patient was on beta-blocker and ACE inhibitor; one patient, ACE inhibitor and diuretics; and one patient, beta-blocker alone. The median age at which surgical repair was performed was 3.7 years (IQR 2.3-6.9) and the median time from surgical repair was 21.4 years (IQR 16.4-26.4). Ten patients (27.8%) had undergone transannular patch repair (Table 2). All patients were in sinus rhythm.

**Table 2:** **Medication, Surgical and CMR Data of TOF patients (n = 36)**

	TOF (n = 36)
**Medication data**	
Beta-blockers	2 (5.6%)
ACE Inhibitors	2 (5.6%)
Diuretics	1 (2.8%)
Digoxin	0
**Surgical data**	
Age of surgical repair	3.69 (2.26, 6.93)
Time from surgical repair	21.4 (16.4, 26.4)
Transannular patch^*^	10 (27.8)
Pulmonary outflow patch^*^	4 (11.1)
RV to pul artery conduit^*^	4 (11.1)
Previous palliative shunt	4 (11.1)

Median and interquartile ranges are reported for continuous data and frequency and percentage for categorical data.

*Complete surgical data missing in 6 patients.

**Figure 1:**
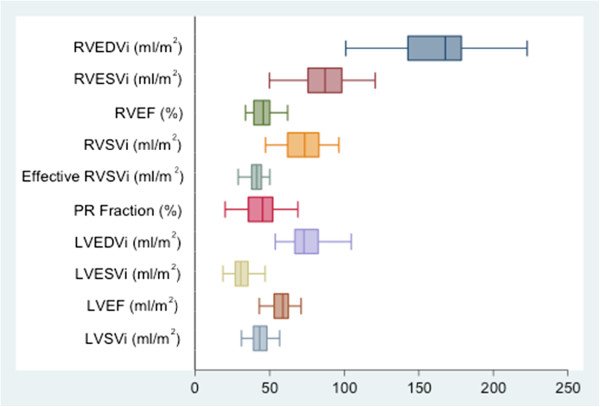
**Box plot showing distribution of CMR data.**

The median RVEDVi, RVESVi, RVEF, RVSVi, effective RVSVi and PR fraction were 167.7 ml/m^2^ (IQR 142.5-178.5), 87.2 ml/m^2^ (IQR 75.8-98.4), 45.6% (IQR 39.2-50.0), 73.5 ml/m^2^ (IQR 61.8-82.9), 41.1 ml/m^2^ (37.9-44.3) and 45.0% (IQR 35.5-52), respectively. The median LVEDVi, LVESVi, LVEF and LVSVi were 73.1 ml/m^2^ (IQR 66.6-82.5), 30.4 ml/m^2^ (IQR 26.9-35.3), 58.7% (IQR 52.9-62.3) and 43.1 ml/m^2^ (IQR 39.0-48.1), respectively. All patients had LVEF >40%, with five (13.9%) patients having LVEF between 40-50%. Twenty-six (72.2%) had restrictive RV physiology; and 20 (55.6%), RVOT aneurysms (Figure 1).

### Relationship between CPET and CMR parameters in TOF patients

**Figure 2:**
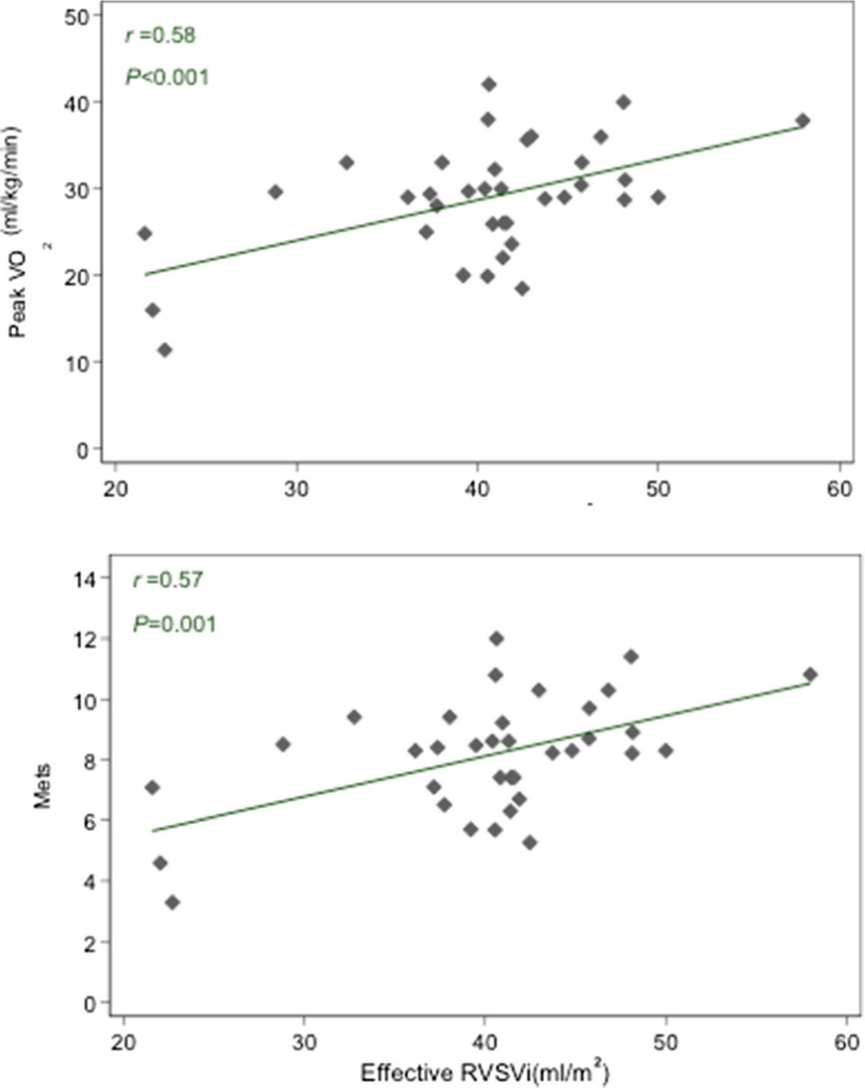
**Scatter plots with linear fit showing relationship between CPET and CMR parameters (r and P value were adjusted for age and gender).**

The median time between CPET and CMR was 2.0 months (IQR 0.8-7.2); there was no change in clinical status between CPET and CMR for all patients. Larger RVEDVi (*r* = 0.36, 95% CI 0.03-0.62, p = 0.035), RVESVi (*r* = 0.35, 95% CI 0.01-0.61, p = 0.042) and LVEDVi (*r* = 0.39, 95% CI 0.06-0.64, p = 0.021) were associated with significantly higher Mets achieved during exercise testing. Larger RVEDVi (r = 0.35, 95% CI 0.03-0.61, p = 0.037) and LVEDVi (*r* = 0.35, 95% CI 0.03-0.61, p = 0.030) were associated with higher peak oxygen consumption achieved during exercise testing. Larger LVSVi (*r* = 0.37, 95% CI 0.04-0.62, p = 0.030) and effective RVSVi (*r* = 0.53, 95% CI 0.24-0.73, p = 0.001) were associated with significantly higher Mets achieved. Larger LVSVi (*r* = 0.35, 95% CI 0.02-0.61, p = 0.037) and effective RVSVi (*r* = 0.53, 95% CI 0.24-0.73, p < 0.001) were associated with significantly higher peak oxygen consumption. Greater PR fraction was associated with lower AT (*r* = -0.40, 95% CI -0.65 to 0.07, p = 0.020)(see Table 3). After adjusting for age and sex, all these relationships remained significant (Table 4, Figure 2). Of note, there was no significant correlation between exercise data and either RVEF or RVSV. Comparing TOF patients with and without restrictive RV physiology, no significant difference in CPET parameters was observed. This was the same among those with and without RVOT aneurysms. There was also no significant association between the rest of the CMR parameters and CPET observations.

**Table 3:** **Relationship between exercise testing and RV and LV function/volume in TOF patients (n = 36)**

CMR parameters	CPET parameters							
	Max breathing reserve	Mets	% predicted mets	Peak oxygen consumption	% Predicted peak oxygen consumption	Anaerobic threshold	Oxygen pulse pressure	VE/VCO _2_ slope
**RVEDVi**	0.15 (-0.20, 0.46)	0.36 (0.03, 0.62)*	0.31 (-0.04, 0.59)	0.35 (0.03, 0.61)*	0.24 (-0.10, 0.52)	-0.03 (-0.36, 0.31)	-0.02 (-0.35, 0.31)	-0.15 (-0.46, 0.20)
**RVESVi**	0.25 (-0.10, 0.54)	0.35 (0.01, 0.61)*	0.28 (-0.07, 0.57)	0.31 (-0.02, 0.58)	0.18 (-0.16, 0.48)	0.12 (-0.23, 0.44)	-0.08 (-0.40, 0.26)	-0.16 (-0.47, 0.19)
**RVEF**	-0.31 (-0.59, 0.03)	-0.16 (-0.47, 0.18)	-0.10 (-0.43, 0.25)	-0.12 (-0.43, 0.22)	-0.01 (-0.34, 0.32)	-0.25 (-0.54, 0.10)	0.15 (-0.19, 0.46)	0.09 (-0.26, 0.42)
**RVSVi**	-0.13 (-0.45, 0.22)	0.16 (-0.18, 0.47)	0.18 (-0.17, 0.50)	0.18 (-0.15, 0.48)	0.18 (-0.15, 0.48)	-0.26 (-0.55, 0.09)	0.10 (-0.23, 0.42)	-0.05 (-0.38, 0.29)
**Effective RVSVi**	-0.18 (-0.49, 0.16)	0.53 (0.24, 0.73)**	0.45 (0.13, 0.69)**	0.53 (0.24, 0.73)**	0.53 (0.24, 0.73)**	0.20 (-0.15, 0.50)	0.28 (-0.06, 0.55)	-0.12 (-0.44, 0.23)
**PR Fraction**	0.07 (-0.27, 0.40)	-0.33 (-0.60, 0.004)	-0.24 (-0.54, 0.12)	-0.30 (-0.57, 0.03)	-0.33 (-0.59, 0.002)	-0.40 (-0.65, -0.07)*	-0.18 (-0.48, 0.16)	0.02 (-0.32, 0.36)
**LVEDVi**	0.19 (-0.16, 0.50)	0.39 (0.06, 0.64)*	0.05 (-0.30, 0.38)	0.35 (0.03, 0.61)*	0.11 (-0.23, 0.42)	0.23 (-0.12, 0.53)	-0.02 (-0.35, 0.31)	-0.11 (-0.43, 0.24)
**LVESVi**	0.18 (-0.17, 0.49)	0.27 (-0.07, 0.55)	-0.06 (-0.40, 0.29)	0.23(-0.11, 0.52)	-0.05 (-0.37, 0.28)	0.20 (-0.15, 0.50)	-0.03 (-0.36, 0.30)	-0.02 (-0.36, 0.32)
**LVEF**	-0.07 (-0.40, 0.28)	-0.06 (-0.39, 0.28)	0.13 (-0.22, 0.45)	-0.04 (-0.36, 0.29)	0.19 (-0.15, 0.49)	-0.13 (-0.45, 0.22)	0.04 (-0.29, 0.36)	-0.09 (-0.42, 0.26)
**LVSVi**	0.11(-0.23, 0.43)	0.37 (0.04, 0.62)*	0.18 (-0.17, 0.50)	0.35 (0.02, 0.61)*	0.28(-0.05, 0.56)	0.15 (-0.20, 0.47)	0.01 (-0.32, 0.34)	-0.20 (-0.50, 0.15)

Pearson correlation coefficient, *r*, with 95% CI shown.

*p < 0.05.

**p < 0.01.

**Table 4:** **Correlates of CPET parameters on CMR parameters with multiple linear regression analysis adjusting for age and gender**

CPET	MRI	Adjusted ***r***	95% CI	***P***	***R*** ^2^,%
Mets	RVEDVi	0.41	(0.07, 0.67)	0.019	40.3
	RVESVi	0.35	(0.0004, 0.62)	0.047	37.2
	Effective RVSVi	0.57	(0.28, 0.77)	0.001	51.9
	LVEDVi	0.36	(0.02, 0.63)	0.036	38.1
	LVSVi	0.39	(0.04, 0.65)	0.026	39.2
% predicted Mets	Effective RVSVi	0.42	(0.08, 0.68)	0.017	23.3
Peak oxygen consumption	RVEDVi	0.40	(0.06, 0.65)	0.019	43.7
	Effective RVSVi	0.58	(0.29, 0.77)	<0.001	55.4
	LVEDVi	0.35	(0.003, 0.62)	0.045	41.1
	LVSVi	0.37	(0.04, 0.64)	0.029	42.4
% predicted peak oxygen consumption	Effective RVSVi	0.52	(0.21, 0.73)	0.002	28.2
Anaerobic threshold	PR Fraction	-0.40	-0.66, -0.05	0.023	41.2

## Discussion

Our operated TOF patients with significant PR had near normal exercise capacity, albeit lower than that of normal healthy controls. Although direct comparison is difficult because of differences in cohort characteristics, the exercise capacity in our operated TOF patients with significant PR is fairly similar to a published series [8] and better compared to others [7, 17]. These latter studies included patients with all degrees of PR, unlike our study in which only patients with significant PR on echocardiography were studied with CMR and CPET. In clinical practice, only patients with significant PR routinely undergo surveillance cardiac MR. Our study thus provides clinically-relevant data in the subset of patients with significant PR.

We demonstrated a negative correlation between PR fraction and AT, implying a possible deleterious effect on exercise capacity of worsening PR per se. The AT is decreased in patients with heart disease and is an important functional limit: physiological responses to exercise are different above and below the AT [18]. While low AT in heart failure patients is associated with increased mortality [19], the prognostic value of AT in TOF patients has not been previously studied.

In our study, we found that higher effective RV stroke volume correlated significantly with higher Mets and peak oxygen consumption. In contrast, we found no significant correlation between exercise capacity and either RVEF or RV stroke volume, which is in keeping with reported studies [14, 20]. In our TOF cohort, which has similar distribution of RV and LV volume and ejection fraction parameters to that of other studies [14, 21], the majority had normal LV function. Conceivably, the main determinant of cardiac output (and thereby exercise capacity) would thus be LV preload. In patients with significant PR, LV preload may best be assessed by effective RVSVi as LV preload is determined by the actual net RV forward flow, taking into account the degree of PR.

That LV preload may play an important role in determining exercise capacity is further supported by the observation that both RV and LV end-diastolic volumes - both indirect estimates of LV preload - showed positive correlation with Mets achieved and peak VO2, albeit not as strong a correlation as effective RVSVi. This appears to be counter-intuitive to the commonly held notion that increasingly dilated ventricular volumes equates with worsening exercise capacity. However, our results are by no means unique. There was a trend towards higher peak VO2 with increasing biventricular diastolic volumes in another study [20]. Similar to our findings, a recent study of 55 operated TOF patients found significant positive correlation between LVEDVi and peak work achieved [14]. They also found that exercise capacity between patients with and without RV dilation was similar. Supporting this notion, another study comparing treated pulmonary stenosis and TOF patients found similar exercise capacities and biventricular function in both groups, despite worse PR and larger RV volumes in TOF patients [22]. Our observations, as well as as those of other investigators, call into question the presumption that RV dilation necessarily means impaired exercise tolerance.

Questions have been raised regarding the use of RV size as a primary parameter in deciding the timing of PVR. Studies have found that exercise capacity and stroke volume are preserved late after tetrology repair, despite severe right ventricular dilation [14]. There are several postulations for this. Firstly, this may represent adaptive LV and RV remodeling in well-compensated TOF subjects. Of note, our TOF subjects had near-normal exercise capacities, indicating a good degree of compensation, despite their significant PR. Furthermore, our study has shown that exercise capacity correlates with stroke volumes, supporting the notion that proper adaptation to volume overload of RV could be helpful for preservation of functional capacity. This phenomenon is well recognized in left ventricular physiology among elite athletes [23, 24]. Secondly, PR results in a dilated but not hypertrophied RV. RV dilation exerts lesser impact on oxygen saturation and LV function, so long as RV stroke volume is maintained. Hence, exercise capacity is relatively well preserved [14]. This notion is supported by our observation that effective RV stroke volume is a significant determinant of exercise capacity. The potential of effective RVSVi as an indirect indicator of LV preload, and therefore cardiac output (in patients with largely normal LV function), raises the possibility of this parameter being used for decisions regarding the optimal timing of PVR.

In our study, we have shown that effective RVSVi significantly correlates with exercise capacity and maybe a potentially useful additional parameter in the decision for PVR. Nevertheless, the decision for timing of PVR is a complex one and should not be based primarily on a single parameter. Rather, each case should be considered individually: balancing the need to intervene before the point of irreversible remodeling against the likelihood for repeat procedures in future. The use of ventricular volumes alone may not take into account the degree of compensation of ventricular function. Many factors may also influence the poorer exercise performance in operated TOF patients: not only residual PR, but also residual right ventricular outflow tract obstruction, impaired biventricular function and presence of ventricular or atrial arrhythmias [25].

Abnormal cardiac function and and haemodynamic abnormalities secondary to residual PR and other residual defects may only appear after longer periods of follow-up. As time passes, PR leads eventually to worsening right ventricle enlargement and dysfunction, with consequent biventricular dyssynchrony, progression to heart failure, and poor performance at physical exercise. The challenge is to identify the optimal timing prior to irreversible decompensation for intervention. Presence of arryhythmia is another important consideration in the decision for PVR. Ventricular and atrial arrhythmias occur not uncommonly after correction of TOF and are one of the causes for sudden cardiac death in this group of patients [1, 26]. Both the ESC and CCS guidelines recommend PVR in the presence of sustained atrial or ventricular arrhythmias [10, 11]. In our cohort of repaired TOF patients, none had any malignant arrhythmias necessitating PVR.

The strength of our study lies in the unique set of data on exercise capacity in operated TOF patients with significant PR, which provides insights into the relationship between exercise capacity and CMR findings in this group of patients. The main limitations of our study is its retrospective and cross-sectional design, with lack of longitudinal CPET and CMR data. The longitudinal relationship between exercise capacity and biventricular volumes and function were not addressed and are best studied in a prospective cohort. The retrospective nature of our study also did not allow us to collect data on exercise habits and behavior which may be significant in this group of young patients and could better help explain our findings. Further, most subjects had near normal exercise capacity, which limits extrapolation of the findings to TOF cases with end-stage symptoms. Finally, our study had been performed in a single centre with relatively few patient number. This could have inadvertently introduced some bias and affect the generalizability of our results.

## Conclusion

Although the majority of our TOF subjects had near normal exercise capacity; objective measures of Mets achieved, VO_2_max and peak oygen pulse were significant lower than controls. Increased PR fraction in TOF patients was associated with lower AT. Indexed effective RV stroke volume, which we postulate to be a measure of LV preload in the presence of significant PR, was positively associated with VO_2_max and Mets achieved. This may potentially be used as a predictor of exercise capacity in repaired TOF with significant PR.

## Abbreviations

TOF: Tetralogy of Fallot
RV: Right ventricle
CPET: Cardiopulmonary exercise testing
CMR: Cardiac magnetic resonance imaging
ACC?AHA: American College of Cardiology/American Heart Asscoiation
ESC: European Society of Cardiology
CCS: Canadian Cardiovascular Society
PVR: Pulmonary valve replacement
PR: Pulmonary regurgitation
EDV: End diastolic volume
ESV: End systolic volume
i: indexed
LV: Left ventricle
RVOT: Right ventricular outflow tract
EF: Ejection fraction
SV: Stroke volume
VO2: Oxygen uptake
VE: Minute ventilation
VCO2: Carbon dioxide output
AT: Anaerobic threshold
CI: Confidence interval.
